# Decreased expression of endogenous feline leukemia virus in cat lymphomas: a case control study

**DOI:** 10.1186/s12917-015-0378-9

**Published:** 2015-04-10

**Authors:** Milica Krunic, Reinhard Ertl, Benedikt Hagen, Fritz J Sedlazeck, Regina Hofmann-Lehmann, Arndt von Haeseler, Dieter Klein

**Affiliations:** Center for Integrative Bioinformatics Vienna, Max F. Perutz Laboratories, University of Vienna, Medical University of Vienna, A-1030 Vienna, Austria; VetCore Facility for Research, University of Veterinary Medicine Vienna, A-1210 Vienna, Austria; Bioinformatics and Computational Biology, Faculty of Computer Science, University of Vienna, A-1090 Vienna, Austria; Clinical Laboratory, and Center for Clinical Studies, Vetsuisse Faculty, University of Zurich, CH-8057 Zurich, Switzerland

**Keywords:** Lymphoma, Cats, Feline leukemia virus, Next-generation sequencing

## Abstract

**Background:**

Cats infected with exogenous feline leukemia virus (exFeLV) have a higher chance of lymphoma development than uninfected cats. Furthermore, an increased exFeLV transcription has been detected in lymphomas compared to non-malignant tissues. The possible mechanisms of lymphoma development by exFeLV are insertional mutagenesis or persistent stimulation of host immune cells by viral antigens, bringing them at risk for malignant transformation. Vaccination of cats against exFeLV has in recent years decreased the overall infection rate in most countries. Nevertheless, an increasing number of lymphomas have been diagnosed among exFeLV-negative cats. Endogenous feline leukemia virus (enFeLV) is another retrovirus for which transcription has been observed in cat lymphomas. EnFeLV provirus elements are present in the germline of various cat species and share a high sequence similarity with exFeLV but, due to mutations, are incapable of producing infectious viral particles. However, recombination between exFeLV and enFeLV could produce infectious particles.

**Results:**

We examined the FeLV expression in cats that have developed malignant lymphomas and discussed the possible mechanisms that could have induced malignant transformation. For expression analysis we used next-generation RNA-sequencing (RNA-Seq) and for validation reverse transcription quantitative PCR (RT-qPCR). First, we showed that there was no expression of exFeLV in all samples, which eliminates the possibility of recombination between exFeLV and enFeLV. Next, we analyzed the difference in expression of three enFeLV genes between control and lymphoma samples. Our analysis showed an average of 3.40-fold decreased viral expression for the three genes in lymphoma compared to control samples. The results were confirmed by RT-qPCR.

**Conclusions:**

There is a decreased expression of enFeLV genes in lymphomas versus control samples, which contradicts previous observations for the exFeLV. Our results suggest that a persistent stimulation of host immune cells is not an appropriate mechanism responsible for malignant transformation caused by feline endogenous retroviruses.

**Electronic supplementary material:**

The online version of this article (doi:10.1186/s12917-015-0378-9) contains supplementary material, which is available to authorized users.

## Background

Endogenous feline leukemia virus (enFeLV) sequences are found in the genomes of domestic cats (*Felis catus*) and related wild cat species [1,2]. These endogenous provirus sequences are transmitted vertically through the germ line and exhibit high similarity to exogenous feline leukemia virus (exFeLV) species, which are part of the genus of gammaretroviruses [3-6]. ExFeLV are enveloped viruses with an RNA genome. The viral genome is composed of two single-stranded positive-sense messenger RNA (+mRNA) chains inside a viral particle. Before replication, the viral genome is converted to DNA and then integrated into the host genome. The genome contains three viral genes necessary for replication, in the following order: 5′-*gag*-*pol*-*env*-3′ [7,8]. On both ends of the viral genome, there are LTR (long terminal repeats), which contain regulatory sequences. Although, transcription and translation of enFeLV proviruses were detected in various tissues and cell lines, no infectious viruses are produced, due to mutations within essential parts of the viral genome [9-11]. However, recombination between enFeLV sequences with exFeLVs can generate infectious virus particles [11-15].

ExFeLV infection has been associated with the emergence of lymphomas in cats. Infected cats have a higher risk for tumor development compared to uninfected [16]. Since exFeLV (as well as enFeLV) is capable of integrating its viral sequences into the host cell’s genome, insertional mutagenesis and subsequent activation of cellular oncogenes by regulatory elements on the viral LTR region is one possible mechanism responsible for malignant transformation by exFeLV [17-19]. Another potential tumorigenic effect of the virus would be the persistent stimulation of immune cells by viral antigens bringing them at risk for transformation [20]. Due to the implementation of vaccination and elimination programs against exFeLV, the infections rates are decreasing in some regions of the world [21,22], while in other regions the prevalence of the virus remains high [23]. However, recent data suggest that increasing numbers of lymphomas are found among virus-negative cats [24-27]. The transcription of enFeLV has been observed in feline lymphomas [28,29], but it is still unclear if enFeLV could be another cause of malignant transformation.

In this study, we examine the potential influence of FeLV expression in cats that have developed lymphomas and discuss the possible mechanisms that could have induced malignant transformation. To achieve that, we first sought to confirm the absence of exFeLV, which would allow an independent evaluation of the effects of enFeLV expression. We then investigated the difference in enFeLV expression between two conditions: non-malignant lymph nodes (control) and feline intestinal lymphoma tissues (tumor samples). Here we applied two methods to determine the transcription of exFeLV and enFeLV: next-generation RNA-sequencing (RNA-Seq) [30] and for validation -reverse transcription quantitative PCR (RT-qPCR) [31]. Previous studies have measured the expression of FeLV using RT-qPCR [32]. This study presents the first investigation of the expression of FeLV for domesticated cats using next-generation sequencing technologies. Using RNA-Seq, it is possible to analyze the transcriptome at a higher resolution, with a larger dynamic range [30].

## Results

### No exFeLV expression detectable by RNA-Seq and RT-qPCR

**Figure 1:**
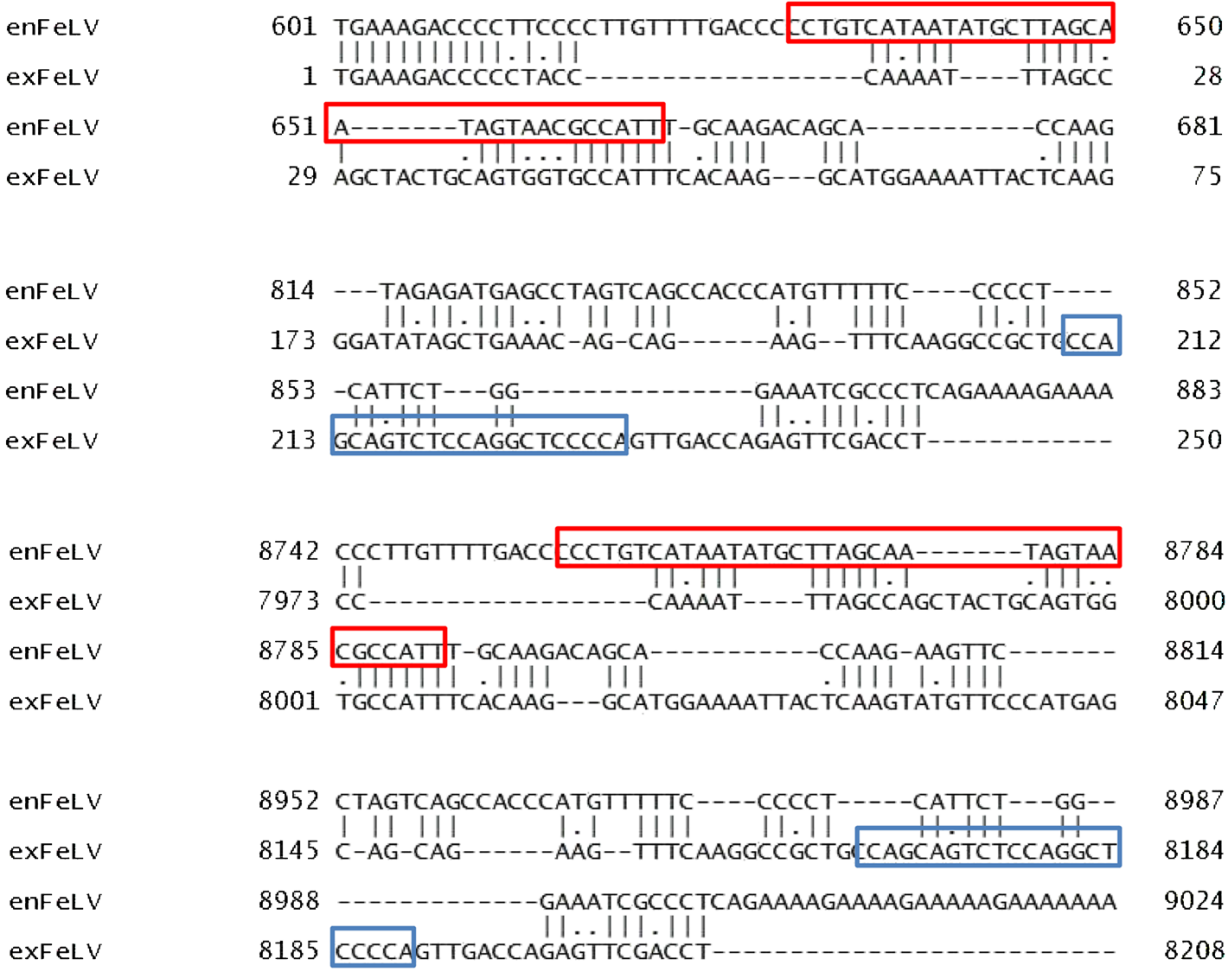
**Pairwise sequence alignment with Needle.** The illustrated viral specific regions are in U3 FeLVs regions. The regions are framed in red and blue colour, which corresponds to enFeLV and exFeLV specific regions, respectively. The numbers present nucleotide position in viral genomes.

The transcriptomes from three control and five tumor cat samples were sequenced. The mean number of sequenced reads in the control samples was 71.33 million, and in the tumor samples was 73.20 million (Additional file 1). We mapped the reads to the reference genomes of both enFeLV and exFeLV (see Methods section for the virus details). As a pairwise sequence alignment reported that the analyzed strains of enFeLV and exFeLV are 74.10% identical, we counted only the reads mapped to virus specific parts of U3 regions in the LTR (Figure 1) to estimate the enFeLV or exFeLV specific expression strength. These virus specific regions were suggested by Tandon et al. [33,34]. Table 1 shows the raw number of mapped reads (MAPQ > 20) to virus specific regions. In control samples, on average 46.33 reads mapped to the enFeLV specific region (35 bp) and in tumor samples, on average 14.80 reads mapped to the same region. In contrast, no reads mapped to the exFeLV specific region (22 bp) in both conditions, indicating that the samples contained only enFeLV viral RNA.

**Table 1:** **RNA**-**Seq and RT**-**qPCR results for expression of exFeLV and enFeLV virus specific regions**

**Condition**	**Cat**	**No. of reads mapped to specific regions** (**MAPQ** ** > ** **20**)		**RT**-**qPCR**			
		**exFeLV probe**	**enFeLV probe**	**enFeLV**	**GAPDH**	**enFeLV/** **1.00 × 10** ^**6**^ **GAPDH**	**exFeLV**
**control**	A	0	24	7.09 × 10^7^	1.04 × 10^8^	6.78 × 10^5^	Not detected
	B	0	83	1.72 × 10^8^	3.43 × 10^8^	5.01 × 10^5^	Not detected
	C	0	32	NA	NA	NA	NA
**tumor**	D	0	4	9.52 × 10^6^	1.37 × 10^9^	6.97 × 10^3^	Not detected
	E	0	11	1.25 × 10^8^	5.33 × 10^8^	2.34 × 10^5^	Not detected
	F	0	16	1.56 × 10^8^	7.61 × 10^8^	2.05 × 10^5^	Not detected
	G	0	16	1.24 × 10^8^	9.78 × 10^8^	1.27 × 10^5^	Not detected
	H	0	27	7.92 × 10^7^	1.22 × 10^9^	6.49 × 10^4^	Not detected

We next used RT-qPCR to confirm the results obtained by RNA-Seq. The tissue samples were investigated for FeLV RNA using the previously illustrated virus specific regions as RT-qPCR probes. Table 1 summarizes the individual results. We detected no exFeLV probe copies among all tested samples, while on average 5.90 × 10^5^ standardized enFeLV probe copies were detected in the control samples vs. 1.28 × 10^5^ that were found in the tumor samples (Table 1).

### Decreased enFeLV expression levels in tumor compared to control samples

We investigated the expression level of the three enFeLV genes (*gag*, *pol* and *env*) for control and tumor samples using RNA-Seq. Table 2 shows the standardized number of mapped reads. On average, 147.84 × 10^−6^ standardized reads mapped to the *gag* gene in the control samples, whereas 40.28 × 10^−6^ standardized reads mapped to the same gene in tumor condition. Thus, a 3.67-fold decrease of *gag* expression was observed in tumor samples. We obtained similar results with the *pol* and *env* genes. On average 152.69 × 10^−6^ standardized reads mapped to the *pol* gene in control and 50.03 × 10^−6^ mapped to the *pol* gene in tumor condition, indicating a 3.05-fold decrease of *pol* gene expression in tumor samples. As for the mapping to *env* gene, we found that in the control condition on average 353.28 × 10^−6^ standardized reads mapped, while on average 98.85 × 10^−6^ standardized reads mapped to the *env* gene in tumor samples, representing a 3.57-fold decrease of *env* expression in tumor compared to control.

**Table 2:** **RNA**-**Seq results for enFeLV genes expression**

**Condition**	**Cat**	**Standardized no. of mapped reads to enFeLV genes** **[x 10** ^**−6**^ ]*		
		**gag**	**pol**	**env**
**control**	A	151.49	132.68	256.50
	B	212.27	244.04	612.54
	C	79.76	81.35	190.80
**tumor**	D	9.91	16.72	22.36
	E	29.54	35.68	71.67
	F	22.90	43.40	105.23
	G	82.13	83.07	135.06
	H	56.90	71.26	159.91

*Standardized by total number of reads (MAPQ > 20).

To test if the expression of each enFeLV gene is significantly different in control compared to tumor condition, we performed three two-sided Mann–Whitney U tests (with the significance level chosen to be 0.05). For the *pol* and *env* genes, tests did not show a significant difference (p-value = 0.07143 for both genes) in the expression between control and tumor samples. For the *env* gene we observed a significant difference in expression (p-value = 0.03571) between the two conditions, towards the control condition. The fold change and the performed tests clearly show that there was no increase in expression in the tumor samples.

We then performed RT-qPCR targeting the enFeLV specific U3 region to confirm the findings from RNA-Seq. The amounts of RT-qPCR detected viral RNA were standardized by the copy numbers of feline GAPDH, which was used as a reference gene for total RNA levels (Table 1). Within all tumor samples, enFeLV RNA copies per 1.00 × 10^6^ copies of GAPDH were found to be lower in comparison to the control samples. When compared to the average enFeLV expression level of control samples, the mean enFeLV expression level in the tumor samples was decreased 4.60-fold. Applying a two-sided Mann–Whitney U test (with the significance level of 0.05), we determined that there was no significant (p-value = 0.09524) difference in the standardized copy numbers of RNA detected from the enFeLV specific U3 region between control and tumor conditions.

## Discussion

### Investigation of exFeLV infection

RNA-Seq analysis showed that there were no reads mapped to the exFeLV specific regions. No evidence of infection with exFeLV was confirmed by RT-qPCR. Additionally, no exFeLV antigen could be detected by a commercial exFeLV ELISA test (SNAP FIV/FeLV Combo Test, Idexx Laboratories).

### EnFeLV transcriptome: control vs. tumor samples

As infections with exFeLV could not be detected among the here tested cats, transcription of the enFeLV in the investigated animals was not affected by interactions with exFeLV, and should have allowed a valid comparison of enFeLV expression levels between tumor and control tissues.

By the means of RNA-Seq: 3.40-fold less viral reads were detected among the tumors compared to control samples. Confirmation of these findings was done by RT-qPCR, where similar values were obtained: the mean enFeLV copies per 1.00x10^6^ GAPDH for all tumor samples turned out to be 4.60-fold lower compared to the mean value of the control samples. It should be noted that the acquired enFeLV expression levels from RNA-Seq were based on the expression of three enFeLV genes while, in contrast, RT-qPCR targeted only the viral U3 region. Mann–Whitney U tests on enFeLV gene counts showed that enFeLV genes transcription in tumor samples was not elevated compared to control tissues, which seems contradictory to observations for the exogenous virus, since a previous study found higher exFeLV viral loads in lymphomas compared to non-malignant tissues [35]. As the exFeLV *env* gene is supposed to have immunosuppressive properties, the increased viral *env* transcription could possibly prime the development and progression of malignancies [36]. In contrast to the exFeLV, our results demonstrate that enFeLV expression levels are not higher on average among all the investigated tumor samples compared to the control tissues. These findings can at least be applied for the here examined exFeLV negative tumors. Nevertheless, one must also take into consideration that a reason for a decreased expression of enFeLV in tumor samples could be the increased transcription of certain cellular transcripts (an increase in the overall mRNA expression is not a rare case for tumors [37,38]), which could lead to the observed decreased proportion of the other mRNA species, including the amount of enFeLV. That would have to be tested by future studies.

In summary, no exFeLV sequences could be detected in the analyzed samples. Although, increased expression of endogenous retroviruses (ERV) has been observed in feline lymphomas [28,29], our data suggest no general increase in the enFeLV transcription levels in lymphoma compared to non-malignant lymphatic tissues. A recent publication investigating human Hodgkin’s lymphoma cells [39] found similar observation of no increase in ERV expression in lymphoma cells compared to normal blood cells. We speculate that the potential impact of enFeLV on the formation of lymphomas seems to be distinct from the exogenous virus. Thus, possible effects of enFeLV on lymphoma development are presumably not due to immunosuppression induced by the expression of viral genes. For enFeLV, high levels of insertional polymorphism have been already described in cats [40]. That led us to believe that insertional mutagenesis of cellular genes by proviral sequences may be a more important mechanism responsible for malignant transformation than viral gene expression induced immunosuppression. However, more data are required to conclusively show that, since other transponsable elements might also play a role in the malignant transformation.

## Conclusions

We show no expression of exFeLV in all analyzed samples. On the contrary, a clear signal indicates the expression of enFeLV in all investigated samples, with no significant increase in enFeLV expression detected in tumor samples compared to control samples. This indicates that the potential tumorogenesis caused by feline endogenous retroviruses cannot be well explained by an immunosuppression mechanism. Further work is necessary to investigate how tumorogenesis in this case occurs.

## Methods

### Animal samples

Tissues of eight domestic cats presented to the clinics of the University of Veterinary Medicine Vienna were included in this study (Table 3). These samples include five lymphoma tissues (tumor samples) and a control group consisting of three lymph nodes. Lymphomas were diagnosed based on routine histopathological examination. Additionally, phenotyping by immunohistochemistry was done for all lymphomas, except for cat D, at which diagnosis was based on histology only. Lymph nodes samples were taken from cats without malignancies that were presented for other diseases: chronic kidney disease (cat A), thromboembolism (cat B) and suspected feline infectious peritonitis (cat C).

**Table 3:** **Sample description**

	**Cat**	**Age**	**Gender**	**Tissue**	**Localization**	**Histological classification**
**control**	A	11 y 6 m	female, neutered	Lymph node	Popliteal	-
	B	4 y 11 m	male, neutered	Lymph node	Mandibular	-
	C	5 y 6 m	female, neutered	Lymph node	Popliteal	-
**tumor**	D	10 y 5 m	female, neutered	Lymphoma	Intestinal	Monomorphic lymphoma
	E	10 y	female, neutered	Lymphoma	Intestinal	Peripheral T-cell lymphoma
	F	8 y 11 m	female, neutered	Lymphoma	Intestinal	Lymphoblastic T-cell lymphoma
	G	5 y 7 m	female, neutered	Lymphoma	Intestinal	Lymphoblastic lymphoma
	H	15 y 2 m	male, neutered	Lymphoma	Intestinal	Diffuse large B-cell lymphoma

### Ethics statement

Animal samples were taken from cats presented to the clinics of the University of Veterinary Medicine Vienna that have been euthanized for clinical reasons. The pet owners agreed to the use of data and sample material for research and educational purposes. The experiments were discussed and approved by the institutional ethics committee in accordance with GSP guidelines and national legislation.

### RNA isolation

All tissue samples were mechanically homogenized on a MagNALyser instrument (Roche Diagnostics, Mannheim, Germany) using 1.4 mm ceramic beads (PeqLab, Erlangen, Germany) at the following settings: 6000 rpm for 30 sec. Subsequently, total RNA was isolated utilizing the RNeasy Mini Kit (Qiagen, Hilden, Germany) according to the manufacturer’s recommendations. Possible contamination with genomic DNA (gDNA) was removed by an on-column DNase I (Qiagen) treatment. RNA quality was investigated by capillary electrophoretic separation of the samples on the Agilent 2100 Bioanalyzer (Agilent Technologies, Santa Clara, CA, USA) and subsequent determination of RNA integrity numbers (RIN). Only samples with a high degree of intact RNA, as determined by RIN-values > 8 were used for further analysis.

### RT-qPCR quantification of enFeLV and exFeLV RNA levels

RNA levels of enFeLV and exFeLV were determined in tissues by RT-qPCR using two virus-specific TaqMan probe assays targeting the U3 regions of enFeLV and exFeLV (enFeLV-U3-1, FeLV-U3-exo) as previously described [33,34]. Viral copy numbers were then standardized to the expression levels of the feline GAPDH gene [41].

### Illumina RNA-sequencing

1 μg total RNA from each sample was used as the starting material for the preparation of cDNA libraries and adjacent RNA-sequencing analysis on a Genome Analyzer IIx system (Illumina Inc., San Diego, CA, USA). Library preparation, including poly-A mRNA purification, and the following next generation sequencing were performed as described in [42], except for the implementation of paired-end sequencing in this study. After 41 sequencing cycles, resulting in one 41-nucleotide (nt) sequencing read per cDNA fragment, a second sequencing round was executed starting from the opposite end of the molecules. Thus, two 41-nt reads were generated for each cDNA fragment revealing the sequence information starting from both ends of the original mRNA template.

### Pairwise sequence alignment

EMBOSS Needle [43] was used to perform and visualize the global sequence alignment between exFeLV and enFeLV. The tool was used with the default parameters, version 6.6.0.

### Mapping and counting RNA-sequencing data

Mapping was done using NextGenMap version 0.4.8. [44]. The non-default parameters were: mode (−m) 1, which uses semi-global alignment for mapping. Identity threshold (−i) was set to 90%. The reads were mapped to the reference consisting of the enFeLV genome [GenBank:AY364318.1] and the exFeLV genome [GenBank:M18247.1].

Mapped reads were filtered for mapping quality MAPQ > 20 using samtools, version 0.1.18[45]. The same tool was used to extract the number of reads mapped to the virus specific regions.

A second mapping using only enFeLV as a reference sequence was performed. Reads were filtered for MAPQ > 20. Using samtools, the number of mapped reads was computed for each viral gene. Standardization was done by dividing the number of mapped reads per viral gene by the total number of reads per sample. To test differential expression strength between control and tumor condition, three two-sided Mann–Whitney U tests (with the significance level chosen to be 0.05) were performed for each of the three enFeLV genes (*gag*, *pol* and *env*). The null hypothesis for the Mann–Whitney U test was that the difference in number of mapped reads between control and tumor condition for a given gene is zero. The alternative hypothesis was that the difference in number of mapped reads per gene between control and tumor condition differs from zero. Since in each test, the same gene was tested, it was not necessary to standardize the number of reads by the length of the gene.

## Additional file

Additional file 1:
**Number of sequenced reads per cat sample.**

## Abbreviations

enFeLV: Endogenous feline leukemia virus
exFeLV: Exogenous feline leukemia virus
LTR: Long terminal repeats
RNA-seq: RNA-sequencing
RT-qPCR: Reverse transcription quantitative PCR

## References

[1] Todaro GJ, Benveniste RE, Callahan R, Lieber MM, Sherr CJ (1975). Endogenous primate and feline type C viruses. Cold Spring Harb Symp Quant Biol.

[2] Roca AL, Pecon-Slattery J, O’Brien SJ (2004). Genomically intact endogenous feline leukemia viruses of recent origin. J Virol.

[3] Mullins JI, Hoover EA, Gallo RC, Wong-Staal F (1990). Molecular aspects of feline leukemia virus pathogenesis. Retrovirus biology and human disease.

[4] Weiss RA (2006). The discovery of endogenous retroviruses. Retrovirology.

[5] Koshy R, Gallo RC, Wong-Staal F (1980). Characterization of the endogenous feline leukemia virus-related DNA sequences in cats and attempts to identify exogenous viral sequences in tissues of virus-negative leukemic animals. Virology.

[6] Song N, Jo H, Choi M, Kim JH, Seo HG, Cha SY (2013). Identification and classification of feline endogenous retroviruses in the cat genome using degenerate PCR and in silico data analysis. J Gen Virol.

[7] Soe LH, Devi BG, Mullins JI, Roy-Burman P (1983). Molecular cloning and characterization of endogenous feline leukemia virus sequences from a cat genomic library. J Virol.

[8] Donahue PR, Hoover EA, Beltz GA, Riedel N, Hirsch VM, Overbaugh J (1988). Strong sequence conservation among horizontally transmissible, minimally pathogenic feline leukemia viruses. J Virol.

[9] Berry BT, Ghosh AK, Kumar DV, Spodick DA, Roy-Burman P (1988). Structure and function of endogenous feline leukemia virus long terminal repeats and adjoining regions. J Virol.

[10] McDougall AS, Terry A, Tzavaras T, Cheney C, Rojko J, Neil JC (1994). Defective endogenous proviruses are expressed in feline lymphoid cells: evidence for a role in natural resistance to subgroup B feline leukemia viruses. J Virol.

[11] Overbaugh J, Riedel N, Hoover EA, Mullins JI (1988). Transduction of endogenous envelope genes by feline leukaemia virus in vitro. Nature.

[12] Pandey R, Ghosh AK, Kumar DV, Bachman BA, Shibata D, Roy-Burman P (1991). Recombination between feline leukemia virus subgroup B or C and endogenous env elements alters the in vitro biological activities of the viruses. J Virol.

[13] Stewart MA, Warnock M, Wheeler A, Wilkie N, Mullins JI, Onions DE (1986). Nucleotide sequences of a feline leukemia virus subgroup A envelope gene and long terminal repeat and evidence for the recombinational origin of subgroup B viruses. J Virol.

[14] Stewart H, Jarrett O, Hosie MJ, Willett BJ (2011). Are endogenous feline leukemia viruses really endogenous?. Vet Immunol Immunopathol.

[15] Anai Y, Ochi H, Watanabe S, Nakagawa S, Kawamura M, Gojobori T (2012). Infectious endogenous retroviruses in cats and emergence of recombinant viruses. J Virol.

[16] Shelton GH, Grant CK, Cotter SM, Gardner MB, Hardy WD, DiGiacomo RF (1990). Feline immunodeficiency virus and feline leukemia virus infections and their relationships to lymphoid malignancies in cats: a retrospective study (1968–1988). J Acquir Immune Defic Syndr.

[17] Bolin LL, Levy LS (2011). Viral determinants of FeLV infection and pathogenesis: lessons learned from analysis of a natural cohort. Viruses.

[18] Fujino Y, Ohno K, Tsujimoto H (2008). Molecular pathogenesis of feline leukemia virus-induced malignancies: insertional mutagenesis. Vet Immunol Immunopathol.

[19] Levy LS, Lobelle-Rich PA (1992). Insertional mutagenesis of flvi-2 in tumors induced by infection with LC-FeLV, a myc-containing strain of feline leukemia virus. J Virol.

[20] Rezanka LJ, Rojko JL, Neil JC (1992). Feline leukemia virus: pathogenesis of neoplastic disease. Cancer Invest.

[21] Weiss AT, Klopfleisch R, Gruber AD (2010). Prevalence of feline leukaemia provirus DNA in feline lymphomas. J Feline Med Surg.

[22] Meichner K, Kruse DB, Hirschberger J, Hartmann K (2012). Changes in prevalence of progressive feline leukaemia virus infection in cats with lymphoma in Germany. Vet Rec.

[23] Bande F, Arshad SS, Hassan L, Zakaria Z, Sapian NA, Rahman NA (2012). Prevalence and risk factors of feline leukaemia virus and feline immunodeficiency virus in peninsular Malaysia. BMC Vet Res.

[24] Hartmann K (2011). Clinical aspects of feline immunodeficiency and feline leukemia virus infection. Vet Immunol Immunopathol.

[25] Louwerens M, London CA, Pedersen NC, Lyons LA (2005). Feline lymphoma in the post-feline leukemia virus era. J Vet Intern Med.

[26] Sparkes AH (1997). Feline leukaemia virus: a review of immunity and vaccination. J Small Anim Pract.

[27] Stutzer B, Simon K, Lutz H, Majzoub M, Hermanns W, Hirschberger J (2011). Incidence of persistent viraemia and latent feline leukaemia virus infection in cats with lymphoma. J Feline Med Surg.

[28] Busch MP, Devi BG, Soe LH, Perbal B, Baluda MA, Roy-Burman P (1983). Characterization of the expression of cellular retrovirus genes and oncogenes in feline cells. Hematol Oncol.

[29] Niman HL, Stephenson JR, Gardner MB, Roy-Burman P (1977). RD-114 and feline leukaemia virus genome expression in natural lymphomas of domestic cats. Nature.

[30] Wilhelm BT, Landry JR (2009). RNA-Seq-quantitative measurement of expression through massively parallel RNA-sequencing. Methods.

[31] Klein D (2002). Quantification using real-time PCR technology: applications and limitations. Trends Mol Med.

[32] Torres AN, O’Halloran KP, Larson LJ, Schultz RD, Hoover EA (2008). Development and application of a quantitative real-time PCR assay to detect feline leukemia virus RNA. Vet Immunol Immunopathol.

[33] Tandon R, Cattori V, Willi B, Meli ML, Gomes-Keller MA, Lutz H (2007). Copy number polymorphism of endogenous feline leukemia virus-like sequences. Mol Cell Probes.

[34] Tandon R, Cattori V, Gomes-Keller MA, Meli ML, Golder MC, Lutz H (2005). Quantitation of feline leukaemia virus viral and proviral loads by TaqMan real-time polymerase chain reaction. J Virol Methods.

[35] Helfer-Hungerbuehler AK, Cattori V, Boretti FS, Ossent P, Grest P, Reinacher M (2010). Dominance of highly divergent feline leukemia virus A progeny variants in a cat with recurrent viremia and fatal lymphoma. Retrovirology.

[36] Lafrado LJ, Lewis MG, Mathes LE, Olsen RG (1987). Suppression of in vitro neutrophil function by feline leukaemia virus (FeLV) and purified FeLV-p15E. J Gen Virol.

[37] Rubie C, Kempf K, Hans J, Su T, Tilton B, Georg T (2005). Housekeeping gene variability in normal and cancerous colorectal, pancreatic, esophageal, gastric and hepatic tissues. Mol Cell Probes.

[38] Valente V, Teixeira SA, Neder L, Okamoto OK, Oba-Shinjo SM, Marie SK (2009). Selection of suitable housekeeping genes for expression analysis in glioblastoma using quantitative RT-PCR. BMC Mol Biol.

[39] Kewitz S, Staege MS (2013). Expression and Regulation of the Endogenous Retrovirus 3 in Hodgkin’s Lymphoma Cells. Front Oncol.

[40] Roca AL, Nash WG, Menninger JC, Murphy WJ, O’Brien SJ (2005). Insertional polymorphisms of endogenous feline leukemia viruses. J Virol.

[41] Kessler Y, Helfer-Hungerbuehler AK, Cattori V, Meli ML, Zellweger B, Ossent P (2009). Quantitative TaqMan real-time PCR assays for gene expression normalisation in feline tissues. BMC Mol Biol.

[42] Ertl R, Klein D (2014). Transcriptional profiling of the host cell response to feline immunodeficiency virus infection. Virol J.

[43] Rice P, Longden I, Bleasby A (2000). EMBOSS: the European Molecular Biology Open Software Suite. Trends Genet.

[44] Sedlazeck FJ, Rescheneder P, von Haeseler A (2013). NextGenMap: fast and accurate read mapping in highly polymorphic genomes. Bioinformatics.

[45] Li H, Handsaker B, Wysoker A, Fennell T, Ruan J, Homer N (2009). The Sequence Alignment/Map format and SAMtools. Bioinformatics.

