# Supplementary material for: Decreased expression of endogenous feline leukemia virus in cat lymphomas: a case control study
Source: BMC Vet Res. 2015 Apr 10;11:90. doi: 10.1186/s12917-015-0378-9 (PMC4424575; doi:10.1186/s12917-015-0378-9)
Supplement: Additional file 1: — Number of sequenced reads per cat sample. [file 12917_2015_378_MOESM1_ESM.pdf]

Additional Table 1

Number of sequenced reads per cat sample

Condition	Cat	Total no. of reads (x 10^6)
control	A	75
	B	71
	C	68
tumor	D	79
	E	73
	F	70
	G	72
	H	72
